# Effective Group Training for Patients with Unexplained Physical Symptoms: A Randomized Controlled Trial with a Non-Randomized One-Year Follow-Up

**DOI:** 10.1371/journal.pone.0042629

**Published:** 2012-08-07

**Authors:** Lyonne N. L. Zonneveld, Yanda R. van Rood, Reinier Timman, Cornelis G. Kooiman, Adriaan van't Spijker, Jan J. V. Busschbach

**Affiliations:** 1 Department of Medical Psychology, Academic Medical Center, University of Amsterdam, Amsterdam, The Netherlands; 2 Department of Anesthesiology, Academic Medical Center, University of Amsterdam, Amsterdam, The Netherlands; 3 Department of Medical Psychology and Psychotherapy, Erasmus Medical Center, Rotterdam, The Netherlands; 4 Department of Psychiatry, Leiden University Medical Centre, Leiden, The Netherlands; 5 Department of Psychotherapy, Riagg Rijnmond, Schiedam, The Netherlands; The James Cook University Hospital, United Kingdom

## Abstract

**Background:**

Although cognitive-behavioral therapy for Unexplained Physical Symptoms (UPS) is effective in secondary care, studies done in primary care produced implementation problems and conflicting results. We evaluated the effectiveness of a cognitive-behavioral group training tailored to primary care patients and provided by a secondary community mental-health service reaching out into primary care.

**Methodology/Principal Findings:**

The effectiveness of this training was explored in a randomized controlled trial. In this trial, 162 patients with UPS classified as undifferentiated somatoform disorder or as chronic pain disorder were randomized either to the training or a waiting list. Both lasted 13 weeks. The preservation of the training's effect was analyzed in non-randomized follow-ups, for which the waiting group started the training after the waiting period. All patients attended the training were followed-up after three months and again after one year. The primary outcomes were the physical and the mental summary scales of the SF-36. Secondary outcomes were the other SF-36-scales and the SCL-90-R. The courses of the training's effects in the randomized controlled trial and the follow-ups were analyzed with linear mixed modeling. In the randomized controlled trial, the training had a significantly positive effect on the quality of life in the physical domain (Cohen's d = 0.38;p = .002), but this overall effect was not found in the mental domain. Regarding the secondary outcomes, the training resulted in reporting an improved physical (Cohen's d = 0.43;p = 0.01), emotional (Cohen's d = 0.44;p = .0.01), and social (Cohen's d = 0.36;p = 0.01) functioning, less pain and better functioning despite pain (Cohen's d = 0.51;p = <0.001), less physical symptoms (Cohen's d = −.23;p = 0.05) and less sleep difficulties (Cohen's d = −0.25;p = 0.04) than time in the waiting group. During the non-randomized follow-ups, there were no relapses.

**Conclusions/Significance:**

The cognitive-behavioral group training tailored for UPS in primary care and provided by an outreaching secondary mental-health service appears to be effective and to broaden the accessibility of treatment for UPS.

**Trial Registration:**

TrialRegister.nl NTR1609 <rctview.asp&quest;TC&hairsp;&equals;&hairsp;1609>

## Introduction

The estimated prevalence of Unexplained Physical Symptoms (UPS) ranges from 18 to 74% in primary care [1], [2], [3], and from 30 to 52% in secondary care [4], [5], [6], [7]. UPS is more prevalent in women than in men [7], [8], [9], [10] and women in their forty's seem to run a higher risk [2], [10]. Other demographic characteristics seemed not be associated with UPS in a consistent manner. For example, some studies found lower socioeconomic background to be associated with UPS [9], [10], while others found an association with having work and a higher education attainment [7]. Patients with UPS attributed their physical symptoms more to physical causes than to lifestyle factors in comparison to patients with a medical diagnosis [7]. Moreover, patients with

UPS are more reluctant than patients with mental disorders to accept a psychiatric diagnosis for their symptoms [11]. UPS are associated with more concomitant psychological symptoms, more impaired functioning and had higher medical utilization than other patient groups [8], [10], [12].

Cognitive-behavioral therapy has shown to be most effective for patients with UPS. It reduces UPS and concomitant psychological symptoms, improves daily functioning, and reduces financial expenses [13], [14], [15] without causing harmful effects [16]. However, the effect of this treatment has been studied mainly in medical subspecialty clinics or mental health centers [13], [17], [18] – resources that are not easily accessible to patients [19], either because their capacity is limited, or because patients refuse to be referred to the mental-health services [17], [20].

To make treatment for UPS more accessible to patients, general practitioners have been trained to carry out cognitive-behavioral therapy. However, only two studies have shown effect when this therapy was provided by general practitioners [21], [22]; most other studies were unable to show any conclusive effect [23], [24], [25], [26], [27], [28]. Also, the transfer of this therapy by general practitioners into routine clinical practice has been hampered by practical issues at the level of the general practitioner, the patients and the treatment. At the level of general practitioners, the implementation was difficult as they hesitated to implement this treatment for UPS. In a British study [29], 1,934 general practitioners were invited to be trained in cognitive-behavioral therapy. Despite the promise of financial compensation, only 70 agreed to participate (3.6%). Those who did participate reported difficulties in implementing the therapy in their family practice because of, for example, the limited time available in patient-physician encounters [30]. At patient's level, the implementation was difficult as patients hesitated to disclose psychosocial issues to their general practitioners [31] and were less satisfied about the quality of care from their general practitioners than patients with a medical diagnosis. For example, they felt that the general practitioner did not take them seriously and took too little time for them [12], [32]. At treatment level, the implementation was difficult as general practitioners and patients had different objectives for their encounters: the former aimed to explain and alleviate symptoms, while the latter hoped to find clinician support [31], [33].

As an alternative to training general practitioners to carry out cognitive-behavioral therapy for UPS, it might be possible for professional therapists from a secondary community mental-health service to make this treatment easily accessible to primary-care patients. First, however, three problems should be resolved: the capacity of secondary care should be increased, patients' refusal to be referred to mental-health services should be reduced, and therapists' and patients' objectives for treatment encounters should be aligned.

As a secondary community mental-health service, we approached these problems as follows. First, to increase capacity, we organized group treatment instead of individual treatments. Second, to minimize patients' refusal to be referred to mental-health services, we sought close collaboration with medical services and offered treatment locally at their centers. Moreover, we used a cognitive-behavioral model which had previously achieved high acceptance in a secondary medical outpatient clinic [34]. As the available manuals based on this model were only intended for individual treatments [35], [36], [37], we had to write a manual for group treatment [38].Third, to align the objectives of the therapists and patients, we tailored the treatment to match primary-care patients' objectives for treatment.

### Objectives

Our first objective was to evaluate the effectiveness of the cognitive-behavioral group training tailored to primary care patients and provided by a secondary community mental-health service reaching out into primary care. The second aim was to observe whether the effect of this group training was preserved in a one-year follow-up period. Our hypotheses were that the group training could raise the quality of life in patients with UPS, and that this effect could be preserved during the follow-up.

## Methods

The protocol for this trial and supporting CONSORT checklist are available as supporting information; see Checklist S1 and Protocol S1.

### Ethics

The study was approved by the Erasmus Medical Research Ethics Committee and was registered in the Dutch Trial Register (NTR 1609) [39]. A detailed description of the study protocol has been published elsewhere [40]. Patients in this study gave written informed consent.

### Study design

The effectiveness of the group training was investigated in a randomized controlled trial. To this end, patients were randomized either to the training or a waiting list after they had completed the baseline measurement (T0). The second measurement (T1) was made directly after the training (13 weeks), or after the same period for those on the waiting list.

The preservation of the effect of the group training was investigated in a non-randomized one-year follow-up. To this end, patients, who had been randomized to the waiting list and had waited, started the training after their second measurement (T1). Patients, who attended the training directly after randomization or after the waiting period, were followed-up three months after the end of treatment (T2), and again one year later (T3).

### Participants

General practitioners and specialists were asked to refer patients aged between 18 and 65 whose physical symptoms, according to their clinical judgment, could not be fully explained by a known medical condition.

Patients were included if they signed the informed consent, and if their UPS fulfilled the DSM-IV criteria for an undifferentiated somatoform disorder or a chronic pain disorder. To verify whether the UPS fulfilled the criteria for undifferentiated somatoform disorder or chronic pain disorder, we used the Structured Clinical Interview for DSM-IV Axis I Disorders (SCID-I) [41], a semi-structured validated interview for making the major DSM-IV Axis I diagnoses.

Patients were excluded from the study if poor language skills or handicaps such as cognitive impairment prevented them from understanding the training.

### Interventions

**Figure 1:**
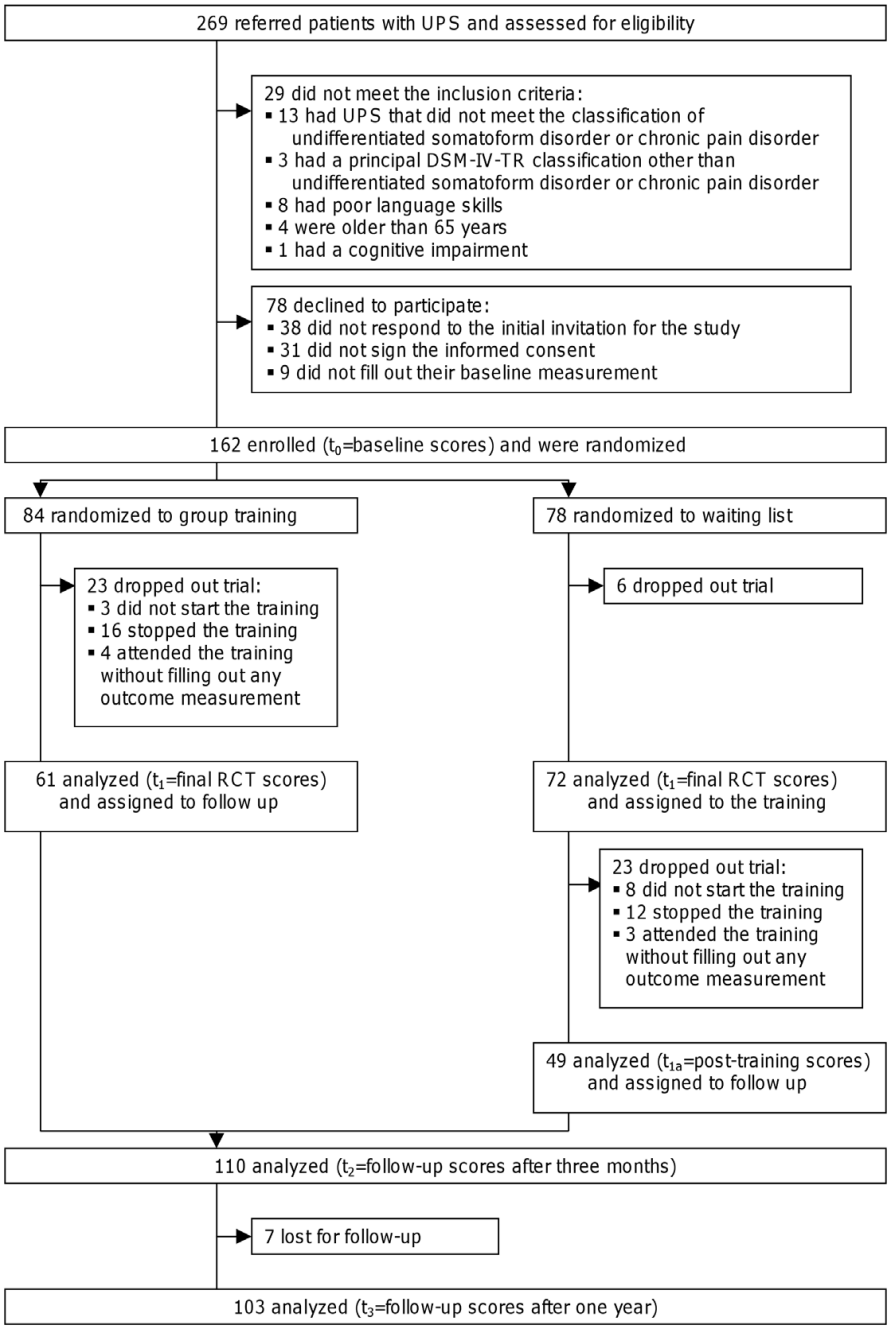
*Tailored* consequences model.

The intervention is a cognitive-behavioral therapy based on the consequences model. In Figure 1, the consequences model is drawn with the solid arrows [42]. In the consequences model, psychological and social factors, which are commonly labeled as causes [43], are labeled as consequences of UPS. UPS (such as abdominal pain) in itself is seen as a stressful condition about which patients develop dysfunctional beliefs (such as ‘I have colon cancer’) that produce cognitive, behavioral, physical, and social consequences. In the short term, these consequences have beneficial effects, either by themselves (such as eating easily digestible food to recuperate), or through interaction with other consequences (such as continuing an activity to distract oneself from the abdominal pain). In the long term, however, these consequences might produce self-perpetuating vicious circles that maintain or aggravate UPS (such as eating less and less, and continuing an activity beyond one's physical limits that leads to more abdominal pain and tiredness). The objective of treatment based on the consequences model is to alleviate symptoms [35], [37], [42].

This model was tailored to primary-care patients. The changes resulting from this tailoring are shown in dotted lines and italics of Figure 1. They can be summarized in terms of three adjustments:

#### 1.) Adding and starting bottom-up next to top-down

Our first change was based upon the fact, that, in the original model, beliefs have a central role. However, the focus on thoughts might not fit the more physically orientated way of viewing and communicating of patients [7], [14], [44], and the need to challenge thoughts in cognitive-behavioral therapy has been questioned lately [45].

In our tailored version of the consequences model, we therefore enter the model bottom-up instead of top-down. Herewith, the consequences rather than the beliefs have a central role. By changing and reducing the consequences, beliefs are addressed indirectly, after which the beliefs can still be addressed directly.

#### 2.) Aggravating instead of maintaining reactions

Our second change was based upon the fact that, in the original model, the consequences can maintain UPS. In our view, patients might translate that as personal blame for causing the continuation of their UPS. This does not match primary-care patients' hope of finding clinician support [31], [33].

In our tailored version of the consequences model, causes are consistently labeled as unknown. The consequences therefore aggravate symptoms rather than maintain UPS. In this way, patients are relieved from blame not only for the cause and existence of UPS, but also for its persistence.

#### 3.) Improvement of quality of life instead of symptom alleviation

Our last change was based upon the fact that, in the original model, the treatment objective is to alleviate symptoms [35], [37], [42]. However, primary-care patients mainly hope to find clinician support [31], [33], followed by improving daily functioning and coping with UPS [33].

In our tailored version of the consequences model, the treatment objective is to improve patients' quality of life not only by preventing aggravation of symptoms but also by increasing daily functioning and coping. This expands the opportunities to support patients' reactions and makes support independent of changes in UPS in itself, since its causes are explicitly labeled as unknown.

Based on this tailored cognitive-behavioral model, a manual was developed for a group training called ‘Coping with the consequences of unexplained physical symptoms’ [38]. This training consists of 13 weekly two-hour sessions organized in local medical settings. Table 1 shows the cognitive-behavioral techniques used in each session.

**Table 1:** The cognitive-behavioral techniques used in each session.

Session	Acquaintance	
**1**	Plenary define personal goals for the training	
	Plenary present the characteristics of own UPS	
**Session**	**Consequence**	**Cognitive-behavioral technique**
**2**	Physical	Psycho-education on physical arousal
		Stopping physical arousal and replacing it with abdominal breathing and relaxation
	Behavioral	Psycho-education on habits
		Stopping potentially harmful habits and replacing them with incompatible beneficial ones, such as:
		-drinking warm herbal tea rather than drinking beer to fall asleep in the evening
		-using skin moisturizer rather than scratching to stop a body itch
**3**	Behavioral	Psycho-education on under-activity, over-activity, and the combination of both
		Stopping under-activity, over-activity, or the combination from them and replacing them with scheduling various activities at a feasible pace with short breaks
	Physical	Rehearsal: abdominal breathing and relaxation
**4**	Emotional	Psycho-education on the meaning of emotions and on the physical arousal they cause
		Recognizing emotions as an important sign that
		-the situation at hand does not correspond with own wishes, needs and expectations
		-the situation asks for change and improvement
		Stopping physical arousal of emotions and replacing it with abdominal breathing and relaxation
	Various	Rehearsal: abdominal breathing and relaxation, and pacing activities
**5**	Beliefs	Psycho-education on beliefs
		Stopping dysfunctional beliefs and replacing them with facts and helpful beliefs using Ellis' ABC scheme
	Various	Rehearsal: abdominal breathing and relaxation and pacing activities
**6**	Physical	Psycho-education on physical fitness
		Improving physical fitness by doing daily a low-cardiac physical activity, extending it by a minute per day, to a target of 60 minutes twice daily
	Various	Rehearsal: abdominal breathing and relaxation, and pacing activities
**7**	Cognitive	Psycho-education on information processing
		Stopping dysfunctional information processing and replacing it with a functional information processing
	Social	Summarizing all consequences of own UPS in a scheme and discussing this scheme with an important and trusted person outside the training
	Various	Rehearsal: abdominal breathing and relaxation, pacing activities and graded exercise
**8–12**	Various	Stopping dysfunctional problem solving and replacing it with functional problem-solving using the five steps of the problem-solving method
		(problem attitude, problem definition, alternative solutions, solution plan, and solution implementation & evaluation)
		Rehearsal: abdominal breathing and relaxation, pacing activities and graded exercise
**Session**	**Relapse prevention**	
**13**	Summarizing all discussed techniques	
	Assembling the techniques applicable for own UPS in a personal First Aid kit	
	Rehearsal: abdominal breathing and relaxation, pacing activities and graded exercise	

The control intervention was a waiting list. The waiting period was as long as the period of the intervention.

### Outcomes

To measure improvement in quality of life, we used the 36-item Medical Outcomes Study Short-Form General Health Survey (SF-36), a validated and reliable self-report questionnaire with 36 questions and fixed-response alternatives for assessing functional health and well-being over the past four weeks [46]. The responses are converted into eight multi-item scales (0–100): ‘Physical functioning’, ‘Role functioning physical’, ‘Bodily pain’, ‘General health’, ‘Vitality’, ‘Social functioning’, ‘Role functioning emotional’, and ‘Mental health’. These scales can be summarized into the ‘Physical component summary’, in which the first four of the above eight scales are weighted most heavily; and into the ‘Mental component summary’, in which the last four of the above eight scales are weighted most heavily [47]. These summaries are transformed into T-scores with a mean of 50 and standard deviation of 10. Higher scores on SF-36 scales indicate a better quality of life.

To measure the intensity of a broad range of psychological problems and psychopathology symptoms, we used the revised 90-item Symptom Checklist (SCL-90-R), a validated and reliable self-report questionnaire with 90 questions and fixed response alternatives for assessing the intensity of symptoms over the past week [48]. The responses are summed up in eight multi-item scales: ‘Phobic anxiety’, ‘Anxiety’, ‘Depression’, ‘Somatization’, ‘Obsessive-compulsiveness’, ‘Interpersonal sensitivity’, ‘Hostility’, and ‘Sleep difficulties’. These scales can be summarized in the ‘Global severity index’. Higher scores on SCL-90 scales indicate a higher number or more severe symptoms.

Primary outcome measures were the ‘Physical component summary’ and the ‘Mental component summary’ of the SF-36. Secondary outcome measures were the individual SF-36 scales and the SCL-90-R scales.

For the SF-36, the manual provides an algorithm to compute the scale scores with a single norm for maximum tolerated percentage of missing items. In this algorithm, a SF-36-scale was only computed, if a patient had completed at least 50% of the items belonging to this SF-36 scale. If this was the case, the patient's available items belonging to the same scale were added up and the resulting sum was divided by the number of available scale items of the same patient. If the number of missing items on a SF-36 scale exceeded the 50% percentages, the data for this scale remained missing.

For the SCL-90-R, the manual provides an algorithm to compute the scales with a norm for the maximum tolerated number of missing items. For the scale ‘Sleep difficulties’, the maximum tolerated number of missing items is one; for the other scales, this maximum is two. By this, the maximum tolerated percentage of missing items of the scales ranges from 67% to 98%. We chose to set the maximum tolerated percentage of missing items for all scales on 75%. In the resulting algorithm, a SCL-90-R scale was only then computed, if a patient had completed at least 75% of the items belonging to this SCL-90-R scale. If this was the case, the patient's available items belonging to the same SCL-90-R scale were added up and the resulting sum was divided by the number of available scale items of the same patient. If the number of missing items on a SCL-90-R scale exceeded this 75% percentage, the data for this scale remained missing.

### Sample size

The sample size required was calculated by power analysis. For power analysis, we applied SPSS version 17 and the mixed-model ANOVA procedure described by Aberson [49]. The repeated-measurement correlation required for the power analysis was estimated on basis of the SF-36 manual [47]. In the manual, a two-weeks test-retest correlation of 0.80 was reported for the SF-36 summary scale ‘Mental component summary’ and 0.89 for the ‘Physical component summary’. Taking into account that a reduction of these correlations should be expected as the time period between two measurements in our study was longer and included the intervention, the correlation was estimated at 0.75. The effect size for the power analysis was estimated at 0.40 based on a review [14], in which the effect sizes for cognitive-behavioral treatments in UPS compared to control conditions centered around .40. These values for correlation and effect size, in combination with an alpha of 0.05 and a beta of 0.20 led to a required sample size of 51 in each group. Adjusted for a dropout of one third, this resulted in a total sample size of 153. The presented procedure to estimate the required sample size deviates from the one described in the original trial protocol [39], as the original power analysis did not match the intended and original statistical analysis plan.

### Randomization—Sequence generation

Patients were assigned to the training or to the waiting list according to a computer generated randomization list. This randomization list was generated just before the start of the next training for enrolled patients who had completed all baseline measurements. As each 13-week training followed the previous one in quick succession and holidays accounting for the only gaps between one training and another, a randomization list was usually generated every other 13 weeks.

### Randomization—Allocation concealment

As the randomization list was generated after the patients were assessed for eligibility and enrolled, allocation was certainly concealed for patients and assessors.

### Randomization—Implementation

The randomization list was generated by an investigator who had no clinical involvement in the trial, and was working in a different building than the buildings where assessment and enrollment were done.

Patients were assessed and enrolled by seven psychologists who had been trained in the SCID-I over several sessions. These psychologists were not involved in other parts of the study or patients' treatment.

Patients were assigned after enrollment according to the randomization list by a psychologist who was not involved in the generation of the randomization list, nor in the assessment and enrollment of patients. Patients were informed about their assignments by a letter posted to their home address.

### Blinding

Patients and trainers could not be blinded for group assignment, as the control condition was a simple waiting list. The data were imported and analyzed after patients had completed the trial.

### Statistical methods

#### Effectiveness of the group training

In the randomized controlled trial the comparability of the patients' baseline-variables between patients who completed the randomized controlled trial and who dropped out were analyzed with the two-tailed t-tests for independent samples for the continuous variables, with the two-tailed Mann-Whitney U-tests for the ordinal variables and with the chi-square tests for the categorical variables. The effects of the training were analyzed with linear mixed modeling.

#### Preservation of the effect of the group training

In the non-randomized, observational follow-up, the comparability of the patients' baseline-variables between patients who could be followed up and who were lost were analyzed with the two-tailed t-tests for independent samples for the continuous variables, with the two-tailed Mann-Whitney U-tests for the ordinal variables and with the chi-square tests for the categorical variables. The preservation of the effect of the training were analyzed with linear mixed modeling.

#### Significance level

All statistical analyses were done with the significance level fixed at 0.05 (two-tailed).

## Results

### Participant flow

**Figure 2:**
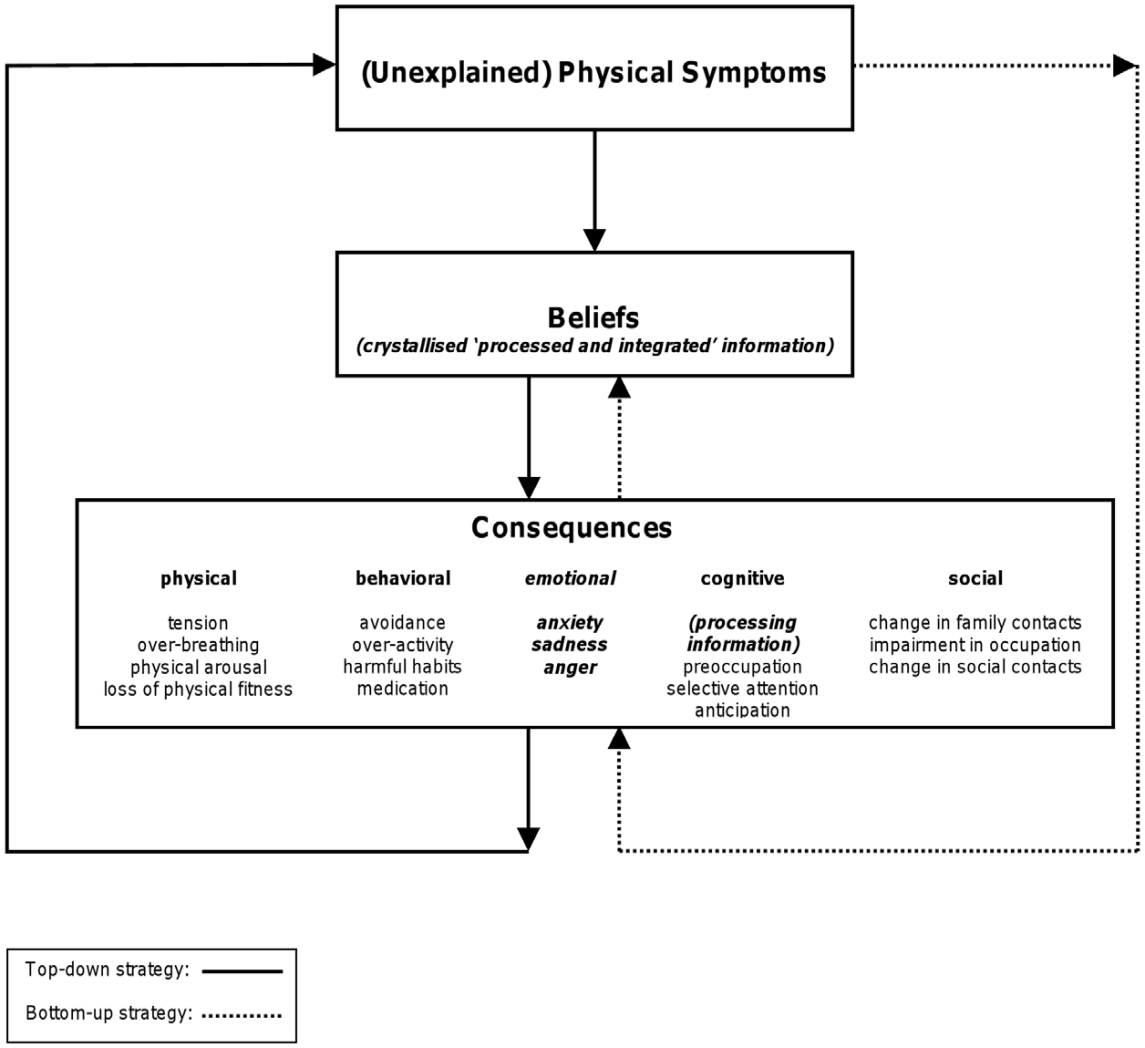
Patient flow. RCT = Randomized Controlled Trial.

Figure 2 shows the flow of patients through the study.

### Recruitment

Patients were recruited between February 2005 and September 2008 in general practices, in outpatient clinics at general hospitals, and by our secondary community mental-health service in and around Rotterdam, the Netherlands. The follow-up ended in December 2009; one year after the intervention group of the last randomization had completed the training.

### Baseline data

Table 2 and Table 3 list the characteristics of the 162 randomized patients, 133 of whom (82%) provided outcome data. There were no significant differences between the 133 patients with primary endpoint outcome data and the 29 who dropped out the randomized controlled trial with regard to the following: UPS characteristics, the number of co-morbid DSM-IV axis I and axis II classifications, referrer characteristics, socio-demographic characteristics, and outcome variables.

**Table 2:** Patients' clinical characteristics.

Clinical characteristics	Group training (n = 84)	Waiting list (n = 78)
**Duration of UPS in years**		
median	8	9.5
interquartile range	3–16	3–17
**Classification of UPS by SCID-I**		
undifferentiated somatoform disorder	32	31
chronic pain disorder	52	47
**Number of comorbid DSM-IV axis I disorders**		
one or more DSM-IV axis I disorders	38	29
**Classification of comorbid DSM-IV axis I disorders**		
mood disorder (lifetime)	13 (40)	11 (30)
anxiety disorder (lifetime)	20 (36)	27 (41)
substance-related disorder (lifetime)	1 (12)	0 (6)
eating disorder (lifetime)	1 (4)	0 (2)
psychotic disorder (lifetime)	0 (0)	0 (1)
somatization disorder	14 (14)	10 (10)
hypochondriasis	1 (1)	1 (1)
adjustment disorder	2 (2)	2 (2)
**Number of comorbid DSM-IV axis II disorders**		
one or more DSM-IV axis II disorders	26	23
Classification of comorbid DSM-IV axis II disorders		
paranoid personality disorder	6	12
schizoid personality disorder	2	3
schizotypal personality disorder	1	1
anti-social personality disorder	0	1
borderline personality disorder	2	5
histrionic personality disorder	1	1
narcissistic personality disorder	0	2
avoidant personality disorder	15	14
dependent personality disorder	2	2
obsessive compulsive personality disorder	14	10
**Referrer**		
primary medical service	41	41
secondary medical service	28	23
secondary mental service	15	14

**Table 3:** Patients' sociodemographic characteristics.

Sociodemographic characteristics	Group training (n = 84)	Waiting list (n = 78)
**Gender**		
female	67	64
male	17	14
**Age in years**		
mean	46	44
interquartile range	38–53	35–52
**Nationality**		
Dutch	72	69
other	12	9
Marital status		
married/living with partner	62	48
unmarried/divorced/widowed	22	30
**Highest education completed**		
primary school or less	7	7
lower vocational or general secondary education	29	25
intermediate vocational or higher general secondary education	33	24
higher vocational, pre-university, or university education	15	21
missing	0	1
**Employment**		
employment	29	28
no employment	55	50

### Intervention data

In total, the training was conducted 20 times in four different local medical settings, with between five and nine patients per intervention (an average of six patients per intervention). The mean attended sessions in the patients who were randomly assigned to the training and provided outcome data was eleven. The minimum number of attended sessions was six.

Each of the 20 groups was led by one of six psychologists with a Master's degree, four of whom had had at least three years' post-Master's experience with group therapy and/or cognitive-behavioral therapy. To compensate for their lack of experience of the other two psychologists, they observed the developer of the manual (LZ) during a group session before jointly leading another group in their own session on the following day. (They gave only one 13-week training.) To increase treatment integrity and positive group dynamics, all psychologists familiarized themselves with the tailored consequences model and the manual's line of reasoning by going through the manual before each session under the supervision of LZ.

### Numbers analyzed

The statistical analyses were conducted according to the intention-to-treat principle [50], as the data of all patients who were randomized were included in the linear mixed modeling.

### Outcomes and estimation

#### Effectiveness of the group training

Table 4 shows the estimates for the effect of the training on the primary and secondary endpoints of the randomized controlled trial. The training had a significant effect on the primary outcome measure ‘Physical component summary’ (p = .002). The effect size was medium (Cohen's d = 0.38) according to Cohen's statistical guidelines [51]. No effect of the training was found for the primary outcome measure ‘Mental component summary’. On the secondary outcome measures of the SF-36, scales in favor of the training indicating significantly better functioning were ‘Role functioning physical’ (Cohen's d = 0.43, p = .01), ‘Bodily pain’ (Cohen's d = 0.51, p = <.001), ‘Social functioning’ (Cohen's d = 0.36, p = .01), and ‘Role functioning emotional’ (Cohen's d = 0.44, p = .01). On the secondary measures of the SCL-90-R, scales in favor of the training indicating a lower number or less severe symptoms were ‘Somatization’ (Cohen's d = −0.23, p = 0.05), and ‘Sleep difficulties’ (Cohen's d = −0.25, p = .04).

**Table 4:** Estimates for training and waiting group.

Scale	Intercept	Time	Time* Training	Training vs. Waiting groep	
Primary endpoint	Estimate [95% CI]	Estimate [95% CI]	Estimate [95% CI]	Cohen's d	p
**SF-36 scale**					
physical component summary	31.2 [30.1–32.8]	1.5 [0.04–3.01]	3.4 [1.3–5.5]	0.38	0.002
mental component summary	45.2 [43.5–46.7]				
**Secondary endpoint**					
**SF-36 scale**					
physical functioning	50.9 [47.1–54.6]	2.2 [−1.3–5.6]	4.7 [−0.3–9.7]	0.19	0.06
role functioning physical	15.6 [11.4–19.8]	5.9 [−0.3–12.1]	11.7 [2.6–20.7]	0.43	0.01
bodily pain	33.2 [30.3–36.2]	0.3 [−3.4–3.9]	9.9 [4.8–15.1]	0.51	<0.001
general health	38.0 [35.2–40.7]	3.8 [1.3–6.4]			
vitality	33.4 [3.06–36.2]	3.4 [−0.4–7.3]	5.4 [0.1–10.7]	0.30	0.05
social functioning	49.2 [45.4–52.9]	1.6 [−3.3–6.6]	8.6 [1.7–15.5]	0.36	0.01
role functioning emotional	72.1 [62.7–81.5]	−10.1 [−20.3–0.11]	18.4 [3.4–33.3]	0.44	0.01
(training group baseline1)	−13.4 [−26.5–−0.4])				
mental health	62.0 [60.1–65.9]				
**SCL-90-R scale**					
phobic anxiety	9.2 [8.6–9.8]				
anxiety	17.6 [16.5–18.7]				
depression	31.7 [30.0–33.5]	−1.9 [−3.4–−0.5]			
somatization	29.2 [27.8–30.5]	−2.0 [−2.6–0.2]	−2.0 [−4.0–0.0]	−0.23	0.05
obsessive-compulsive	20.7 [19.7–21.7]	−1.2 [−2.1–−0.4]			
interpersonal sensitivity	26.7 [25.2–28.2]				
hostility	8.5 [8.0–9.0]				
sleep difficulties	8.0 [7.5–8.6]	−0.4 [−1.0–0.2]	−0.9 [−1.8–0.0]	−0.25	0.04
global severity index	165.5 [158.0–173.0]	−8.1 [−13.3–−2.8]			

Note: Insignificant effects are not presented in this table.

1) Role functioning at baseline was different between training and waiting group.

#### Preservation of the effect of the group training

Table 5 shows the estimates for the effects of time on the primary and secondary endpoints of the non-randomized, observational follow-up. At each time point, time did not eliminate the effects of the training. In contrary, for the primary outcome measure ‘Physical component summary’, the effect increased from Cohen's d 0.39 to 0.49 at three-months follow-up and Cohen's d was still 0.49 at one-year follow-up. A similar trend was observed for the secondary outcome measures ‘Physical functioning’ and ‘Obsessive-compulsive’.

**Table 5:** Estimates at each time point.

Scale	Model estimates				Post training		Three-months follow-up		One-year follow-up	
	Intercept [95% CI]	Time [95% CI]	Time^2^ [95% CI]	Log-time [95% CI]	Estimate [95% CI]	d1)	Estimate [95% CI]	d1)	Estimate [95% CI]	d1)
**Primary endpoint**										
**SF-36 scale**										
physical component summary	31.4 [30.0–32.9]	−0.3 [−0.5–−0.1]		3.3 [2.2–4.4]	3.6 [2.6–4.6]	0.39	4.5 [3.4–5.7]	0.49	4.5 [3.2–5.7]	0.49
mental component summary	44.9 [43.1–46.6]	−0.3 [−0.7–−0.0]		2.2 [0.5–3.9]	2.0 [0.5–3.5]	0.18	2.2 [0.5–3.9]	0.20	0.9 [−1.0–2.8]	0.08
**Secondary endpoint**										
**SF-36 scale**										
physical functioning	51.5 [47.7–55.3]			2.2 [1.1–3.3]	3.1 [1.5–4.6]	0.12	4.3 [2.1–6.5]	0.17	6.1 [3.0–9.2]	0.25
role functioning physical	15.7 [10.7–20.7]	−1.5 [−2.5–−0.5]		13.9 [8.8–18.9]	14.8 [10.1–19.4]	0.45	18.1 [12.7–23.4]	0.56	16.1 [9.2–23.1]	0.50
bodily pain	33.2 [30.2–36.3]	−5.5 [−10.0–−1.1]	0.2 [0.0–0.4]	17.0 [.0–27.1]	8.8 [5.8–11.8]	0.45	7.0 [3.9–10.2]	0.36	8.4 [4.1–12.8]	0.43
general health	37.9 [35.1–40.8]	−0.6 [−1.0–−0.1]		5.5 [30. –80.1]	6.0 [3.7–8.3]	0.33	7.4 [4.8–10.0]	0.40	7.0 [4.1–9.9]	0.38
vitality	33.4 [30.5–36.3]	3.0 [2.1–3.9]	−0.2 [−0.2–−0.1]		7.6 [5.4–9.7]	0.39	12.2 [8.8–15.5]	0.63	8.6 [5.2–12.0]	0.45
social functioning	49.2 [45.4–53.1]	−0.9 [−1.7–−0.2]	8.2 [4.2–12.3]		8.7 [5.0–12.3]	0.35	10.5 [6.4–14.6]	0.42	9.1 [4.5–13.6]	0.36
role functioning emotional	65.6 [60.3–70.8]									
mental health	62.6 [59.7–65.6]	1.1 [0.4–1.8]	−0.1 [−0.1–−0.0]		2.7 [0.9–4.5]	0.14	4.2 [1.4–7.1]	0.22	1.6 [−1.8–5.1]	0.09
**SCL-90-R scale**										
phobic anxiety	9.3 [8.6–9.9]	0.1 [0.0–0.2]		−0.5 [−0.9–−0.1]	−0.4 [−0.8–−0.0]	−0.10	−0.4 [−0.9–0.0]	−0.10	0.0 [−0.6–0.6]	−0.00
anxiety	17.9 [16.8–19.1]	−0.4 [−0.6–−0.2]	0.02 [0.01–0.03]		−0.9 [−1.5–−0.4]	−0.12	−1.5 [−2.3–−0.6]	−0.20	−0.9 [−2.1–0.2]	−0.12
depression	31.8 [30.0–33.5]	0.4 [0.1–0.6]		−3.1 [−4.4–−1.9]	−3.2 [−4.3–−2.0]	−0.28	−3.7 [−5.1–−2.3]	−0.33	−2.8 [−4.7–−0.8]	−0.24
somatization	29.2 [27.9–30.4]	0.4 [0.2–0.6]		−3.0 [−4.0–−2.1]	−3.1 [−4.0–−2.2]	−0.38	−3.8 [−4.8–−2.7]	−0.45	−3.0 [−4.5–−1.6]	−0.36
obsessive-compulsive	20.7 [19.7–21.7]	−0.5 [−0.7–−0.3]	0.02 [0.01–0.04]		−1.3 [−1.8–−0.8]	−0.20	−2.2 [−3.0–−1.4]	−0.34	−2.2 [−3.3–−1.1]	−0.34
interpersonal sensitivity	26.7 [5.1–28.2]									
hostility	8.3 [7.8–8.8]									
sleep difficulties	8.0 [7.5–8.6]	0.12 [0.03–0.21]		−0.9 [−1.4–−0.4]	−0.88 [−1.30–−0.47]	−0.25	−1.02 [−1.50–−0.54]	−0.29	−0.67 [−1.26–−0.07]	−0.19
global severity index	165.5 [158–173]	1.37 [0.45–2.29]		−11.1 [−15.6–−6.61]	−11.3 [−15.4 −7.1]	−0.23	−13.4 [−18.5–−8.3]	−0.27	−10.1 [−17.9–−2.5]	−0.21

Note: Insignificant effects are not presented in this table.

1) d = Cohen's d compared to baseline.

### Ancillary analyses

To explore whether patients who had one-year follow-up scores differed from patients who had no one-year follow-up scores, these two groups were compared with each other with regard to the following: UPS characteristics, the number of co-morbid DSM-IV axis I classifications, referrer characteristics, socio-demographic characteristics, and outcome variables. Patient with one-year follow-up scores were significantly older (M = 46.69, SD = 10.79) and reported significantly more vitality (M = 35.58, SD = 17.56), and significantly less hostility (M = 7.99, SD = 2.68) than patients without one-year follow-up scores (age: M = 42.44, SD = 11.09, p = .02; SF-36 scale ‘Vitality’: M = 29.60, SD = 17.88, p = .04, and SCL-90 scale ‘Hostility’: M = 9.53, SD = 4.78, p = .03).

### Adverse events

One adverse event was reported in this study. After the training, one patient reported rumination about possible death of beloved people, which tired her out. For this patient, psychotherapy was arranged, in which she engaged. Further detailed description of this patient has been published elsewhere [52].

## Discussion

### Interpretation

The effect of a cognitive-behavioral group training on the quality of life was studied in patients with UPS. The training was based on the consequences model tailored to primary-care patients and provided by a secondary community mental-health service reaching out into the primary care. It was found to be effective at improving the physical domain of quality of life, which was the domain patients reported as most burdensome at baseline. This positive effect was preserved during the entire one-year follow-up period. These results are remarkable, as studies have shown that the prognosis of UPS becomes more unfavorable if the duration of UPS is longer [1], [53], [54], [55], or if UPS is classified as a somatoform disorder [53], [56]. In our study group, the median of the duration of UPS was nine years, and UPS had been classified as undifferentiated somatoform disorder or as chronic pain disorder.

Considering these effects, further research on this training seems to be worthwhile.

The effects might differ between various subgroups within patients with UPS. Further research could explore, whether some subgroups benefit more than others. If the latter is the case, allocation and selection might improve effectiveness even more. Also, it would be interesting to explore whether the training also reduces costs by reducing medical utilization, and productivity losses due to UPS. This would make the training not only more interesting from a patient's but also from a societal's perspective.

### Generalizability

Various terms are used for unexplained physical symptoms. Examples of other terms used for these symptoms are Medically Unexplained Physical Symptoms (MUPS), Functional Somatic Symptoms (FSS), abridged somatization, and multisomatoform disorder [3], [11], [57]. The use of different terms and different definitions makes the communication about these symptoms between clinicians and researchers within and between disciplines difficult and reduces generalizability. This might be resolved in the revision of the American Psychiatric Association's Diagnostic and Statistical Manual for Mental Disorders (DSM). Currently, the proposed revision is to classify this symptoms as Somatic Symptom Disorder, which is defined as persistent distressing somatic symptoms in combination with excessive thoughts, feelings, and behaviors in response to these somatic symptoms [58]. Pending this revision, we chose among all frequently used terms the term Unexplained Physical Symptoms (UPS), because this term reflects best that physical, psychological and social causes and effects are not or not easily separated from each other, and are mostly interrelated without a clear starting or finishing point. By using the term UPS, we acknowledged this interrelationships, promoted transparency in the communication to all stakeholders; patients, clinicians and researchers, and followed the recommendation for terminology on these symptoms, which includes “to remove language that is potentially pejorative to patients” [11].

The prevalence of having one or more co-morbid DSM-IV Axis I disorders in our study was 41%. The three most commonly comorbid DSM-IV axis I disorders were anxiety (29%), mood (15%), and somatization (15%) disorder. These prevalences are comparable with earlier findings in patients with UPS. In this patients' group, studies found prevalences of comorbid anxiety and/or depressive disorders in primary care ranging from 26% [2] to 54% [59]. The prevalence of a comorbid anxiety disorder was 17% and the prevalence of a comorbid depressive disorder was also 17% [2]. It is known [60], that patients with UPS have a higher rate of current mood disorder or current anxiety disorder than either healthy controls or patients with phenomenologically similar medical diseases of known organic pathology. Including patients with co-morbid DSM-IV Axis I disorders makes our results generalizable to a wider group of patients with UPS than is usually selected for scientific trials, and more similar to the patient group seen in routine clinical practice.

The prevalence of having one or more personality disorders in our study was 30%. The three most commonly personality disorders were avoidant (17.9%), obsessive compulsive (14.8%), and paranoid (11.1%) personality disorder. These prevalences are in line with earlier findings in this group of patients. Studies found prevalences of personality disorders in UPS ranging from 0% to 88.6% [61], [62], [63], [64], [65], [66], [67], [68], [69], [70]. The most commonly personality disorder for patients with UPS in these studies differed between obsessive compulsive [61], [65], [67], [68], [69], histrionic [62], [64], avoidant [61], [70], dependent [67], [71] and paranoid [66] personality disorder. Our findings on the prevalence of personality disorders were quite similar to the rates reported in the study in patients with chest pain measuring personality disorders using a self-report questionnaire. This study reported a prevalence of 39%, in which three most commonly reported personality disorders were obsessive-compulsive (23.3%), avoidant (13.8%), and paranoid (13.2%) personality disorder [69]. Differences in prevalences of personality disorders between studies might be explained by the use of different instruments for the assessment of personality disorders, but also by the use of different definitions for unexplained physical symptoms (e.g. somatizing patients and somatization disorder). As our definition of unexplained physical symptoms was symptoms fulfilling the DSM-IV criteria for an undifferentiated somatoform disorder or a chronic pain disorder, prevalences in our study might also be slightly lower than in somatoform disorders in general, because studies [61], [62] suggested that both undifferentiated somatoform disorder and chronic pain disorder were less frequently combined with personality pathology than the other somatoform disorders. Due to the use of validated interviews for both the classification of UPS and personality disorders and comparability of our findings with earlier findings, we believe our results to be reliable and generalizable.

The training is theory-based and elaborately described in the manual [38]. It was conducted by six different psychologists with different experience levels at four different local medical settings. This suggests that the training is transferable to different circumstances.

Conducting the training belonged to the daily activities of the psychologists and was financed within the current reimbursement practice. By this, the training could be implemented without research funds. Kathol et al. [72] showed the relevance for this kind for generalizability, as most evidence-based programs integrating mental health services in primary care could not be successfully implemented after completion of the study due to the fact that research funds were not substituted within the current reimbursement practice.

### Limitations

The options for the study were limited by the fact that it was part of daily activities of a secondary community mental-health service. Because patients on the waiting list had to wait only 13 weeks for the group training – the same period as the training itself – the study was deprived of a control condition for the three-month and one-year follow-ups. However, our time frame of follow-up assessments was longer than the usual time frame of intervention studies for UPS that ranged from three to 12 months with a mean of six months [18].

Not only the length of the control condition for the influence of time and other not-intervention-related circumstances, but also the lack of a control condition for the influence of intervention-related aspects was a limitation. Because of this limitation, the measured effects could not be attributed to the specific therapeutic interventions of our training. If a control intervention group (e.g. relaxation, solely psycho-education, self help, individual treatment) had been included, it would have been possible to explore whether the training itself had supplementary effects in comparison to other interventions or individual treatment.

Another limitation of our study is that inter-rater-reliability was not calculated for the *Structured Clinical Interview for DSM-IV Axis I Disorders* (SCID-I), which verified whether patients fulfilled the inclusion criteria for undifferentiated somatoform disorder or chronic pain disorder. This is especially regrettable, as the number of UPS classified as undifferentiated somatoform disorder was lower than the number of UPS classified as chronic pain disorder – the opposite of what was found in a study in Dutch general practices [2]. To clarify this difference, we examined the SCID-I interviews more closely. This showed that, due to the interviewers' or patients' emphasis on pain in the presence of a broad spectrum of symptoms, syndromes such as fibromyalgia or chronic fatigue syndrome had sometimes been misclassified as chronic pain disorder. These misclassifications might have inflated the number of chronic pain disorders at the expense of undifferentiated somatoform disorder.

Not only the comparability of interviews, but also the comparability of the training's sessions in different groups was not measured. As supervision was given by the developer of the manual (LZ) before each session, treatment integrity and comparability were stimulated but they were not verified. If the group sessions had been recorded, they could have been rated by independent raters and treatment integrity could have been verified.

Randomization was used to reach comparability between the patients in the training and patients on the waiting list. Notably, this randomization resulted in an imbalance of distribution of living with or without a partner over the training and the waiting group, although this imbalance was not significant (p = .13). Nevertheless, such imbalance might have influenced the results in favor of the training, as this demographic variable could be seen as in indicator of social support and the ability to have stable relationships.

### Overall evidence

Earlier studies have found that cognitive-behavioral therapy improved physical symptoms, psychological distress, and functional status [14], [15]. The effect sizes for this therapy compared to control conditions centered around .40 [14]. Physical symptoms appeared to be the most responsive [14], [15], although in some studies for specific syndromes, such as chronic fatigue syndrome and fibromyalgia, the opposite was found, and the effect size for psychological distress was larger [14]. Improvement of the physical symptoms could occur whether or not psychological distress was decreased [73]. Preservation of positive effects was observed in 6-months to one-year follow-up assessments [14].

Our findings seemed to be consistent with these earlier studies as they showed a similar improvement of functional status and symptoms, more responsiveness of physical symptoms in comparison to psychological symptoms, and the preservation of these effects over the one-year follow-up period. The effect size of .38 in the randomized controlled trial and the effect size of .49 in the non-randomized one-year follow-up might even be considered to be relatively high, because our training was designed to be easily accessible, and, thereby, might have included more patients with higher resistance to psychological interventions.

For this, the tailoring of the consequences model for primary-care patients might have been essential. It was only after doing so that we discovered that, due to low acceptance [74], and no effectiveness [27], two previous attempts to use the original consequences model in primary care had failed. Although most patients in our study had been referred by medical services, especially by general practitioners, ‘only’ 78 of the 269 (29%) patients did not attend the first appointments (so-called ‘no shows’); this no-show figure was substantially lower than the estimated 50%–80% of patients who refuse to be referred to mental-health services [20]. Patients also seemed to accept the training itself: 65 of the 84 who were randomized to it (77%), and 52 of the 72 who waited for it (72%) really attended the training.

By seeking close collaboration with medical centers and offering treatment at their centers, we might not only have broadened the accessibility of mental-health services for primary-care patients. As physicians rated the problems of getting mental health services for their patients twice as high as the problems of getting other specialty services [19], we might also have simplified the access of mental-health services for physicians.

The success rate of referrals from medical care services to the training might still be improvable. The three most common reasons for failure to seek treatment after referral are 1.) the problem has resolved, 2.) patients need to wait before treatment starts, and 3.) a lack of motivation [44]. With regard to the first reason, it is suggested by the long duration of UPS in our patients, and also by the overall low recovery rate in patients with somatoform disorders [56], that the problem had not resolved. But the second reason – having to wait for treatment – was certainly an issue: those who had to wait, such as those on the waiting list, complained about it. Indeed, some could not bring themselves to wait and left the trial. Therefore, if this training is implemented in routine clinical practice, a short waiting period before treatment starts is advisable. The third reason – lack of motivation to actualize the referral – revealed another area in which there is scope for further improvement. Patients commented that their physicians had suggested that their complaints were ‘all in their heads’. In certain cases, the sense of not being taken seriously made them delay seeking treatment and made them express anger about it at their first appointment. Feeling disrespected is a factor that is known to influence un-notified no-shows [75].

Perhaps the number of successful referrals might be increased if physicians are trained to use the language of the tailored consequences model to explain the goals of the referral and the treatment – without having to do the cognitive-behavioral interventions themselves. Asking them to do these interventions themselves might not be as effective because of the implementation issues mentioned in the introduction, but also because of difference in education between physicians and psychologists, and the low volume of doing psychological treatment for general practitioners in comparison to psychologists. In medicine, it is a well-established fact, that outcomes raise with higher volumes [76].

In short, the cognitive-behavioral group training tailored for UPS in primary care and provided by an outreaching secondary mental-health service appears to be effective and to broaden the accessibility of the treatment of UPS.

## Supporting Information

Checklist S1 **CONSORT Checklist.**
(DOCX)

Protocol S1 **Trial Protocol.**
(PDF)
