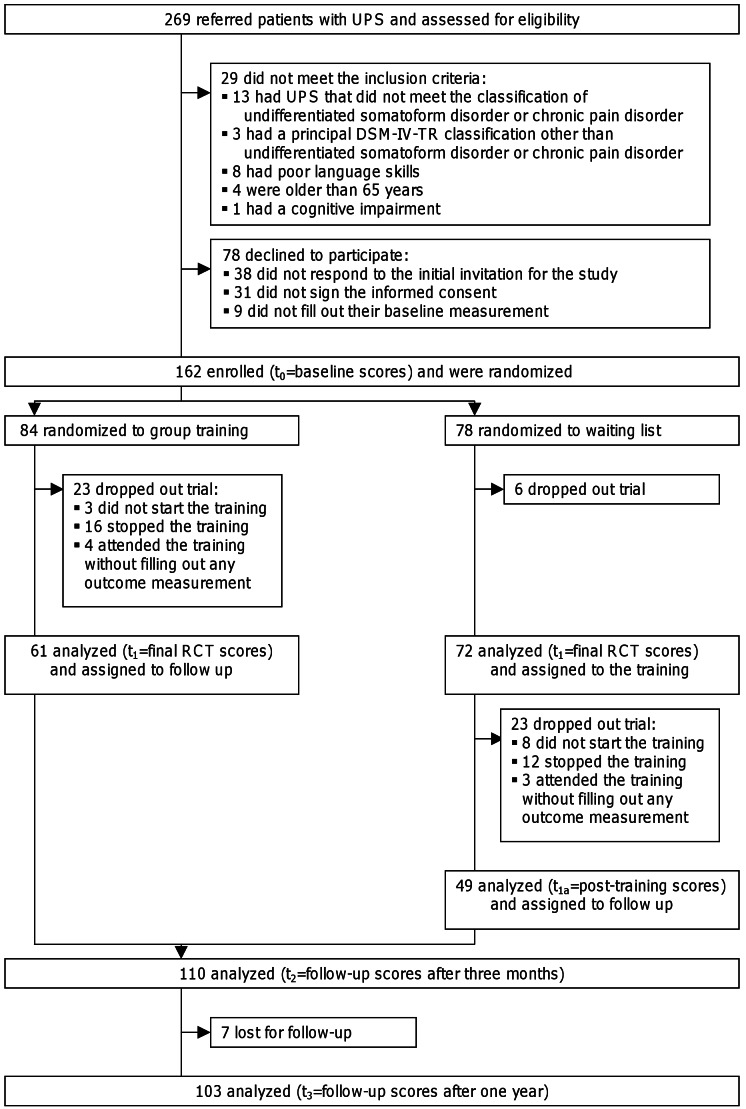# Correction: Effective Group Training for Patients with Unexplained Physical Symptoms: A Randomized Controlled Trial with a Non-Randomized One-Year Follow-Up

**DOI:** 10.1371/annotation/9cefaaa9-c367-4f2d-a927-0a0b304ae21f

**Published:** 2013-05-13

**Authors:** Lyonne N. L. Zonneveld, Yanda R. van Rood, Reinier Timman, Cornelis G. Kooiman, Adriaan van't Spijker, Jan J. V. Busschbach

The locations of Figures 1 and 2 were reversed in the article. The legends are placed correctly. The correct figures are:

Figure 1: 

**Figure:**
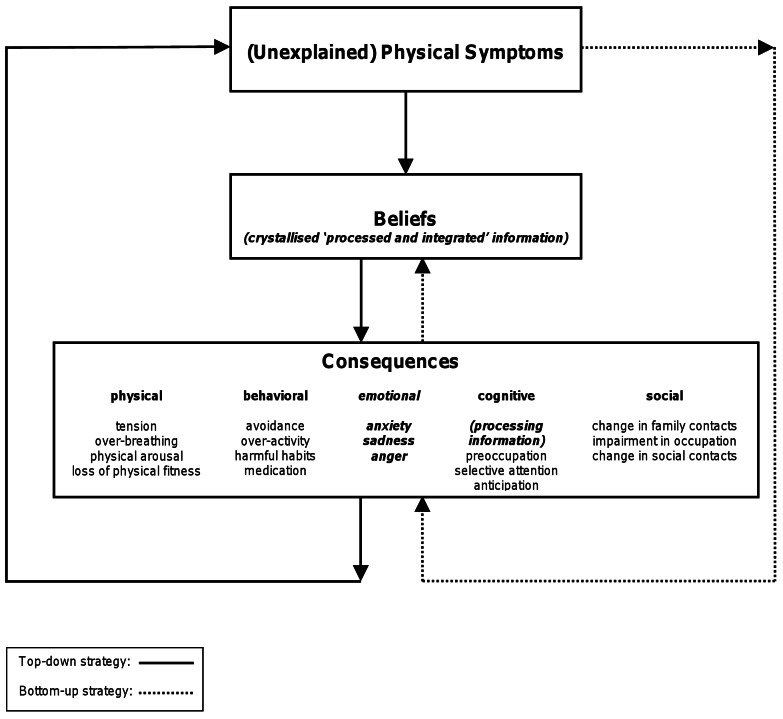


Figure 2: 

**Figure:**