# Supplementary material for: Effective Group Training for Patients with Unexplained Physical Symptoms: A Randomized Controlled Trial with a Non-Randomized One-Year Follow-Up
Source: PLoS One. 2012 Aug 7;7(8):e42629. doi: 10.1371/journal.pone.0042629 (PMC3413637; doi:10.1371/journal.pone.0042629)
Supplement: Protocol S1 — Trial Protocol. (PDF) [file pone.0042629.s002.pdf]

## Protocol S1

The trial protocol has been previously published [1], and has been attached to this document.

1. Zonneveld LNL, Van 't Spijker A, Passchier J, Van Busschbach JJ, Duivenvoorden HJ (2009) The effectiveness of a training for patients with unexplained physical symptoms: protocol of a cognitive behavioral group training and randomized controlled trial. BMC Public Health 9: 251.

BMC Public Health

BioMed Central

Study protocol

Open Access

# The effectiveness of a training for patients with unexplained physical symptoms: protocol of a cognitive behavioral group training and randomized controlled trial

Lyonne NL Zonneveld*[1,2], Adriaan van 't Spijker[2], Jan Passchier[2], Jan J van Busschbach[2] and Hugo J Duivenvoorden[2]

Address: [1]Riagg Rijnmond, Westhavenkade 85, 3133 AV Vlaardingen, the Netherlands and [2]Erasmus Medical Center, Department of Medical Psychology and Psychotherapy, PO Box 2040, 3000 CA Rotterdam, the Netherlands

Email: Lyonne NL Zonneveld* - l.zonneveld@erasmusmc.nl; Adriaan van 't Spijker - a.vantspijker@erasmusmc.nl; Jan Passchier - j.passchier@erasmusmc.nl; Jan J van Busschbach - j.vanbusschbach@erasmusmc.nl; Hugo J Duivenvoorden - h.duivenvoorden@erasmusmc.nl

* Corresponding author

Published: 20 July 2009

*BMC Public Health* 2009, **9**:251 doi:10.1186/1471-2458-9-251

Received: 22 April 2009
Accepted: 20 July 2009

This article is available from: http://www.biomedcentral.com/1471-2458/9/251

© 2009 Zonneveld et al; licensee BioMed Central Ltd.
This is an Open Access article distributed under the terms of the Creative Commons Attribution License (http://creativecommons.org/licenses/by/2.0), which permits unrestricted use, distribution, and reproduction in any medium, provided the original work is properly cited.

## Abstract

**Background:** In primary care, up to 74% of physical symptoms is classified as unexplained. These symptoms can cause high levels of distress and healthcare utilization. Cognitive behavioral therapy has shown to be effective, but does not seem to be attractive to patients. An exception herein is a therapy based on the consequences model, which distinguishes itself by its labeling of psychosocial distress in terms of consequences rather than as causes of physical symptoms. In secondary care, 81% of the patients accepts this therapy, but in primary care the outcome is poor. We assume that positive outcome can also be reached in primary care, when the consequences model is modified and used bottom-up in an easily accessible group training, in which patients are relieved of being blamed for their symptoms. Our aim is to investigate the (cost-)effectiveness of this training.

**Methods and design:** A randomized controlled trial is designed. One hundred patients are randomized to either the group training or the waiting list.

Physicians in general practices and outpatients clinics of general hospitals refer patients. Referral leads to inclusion if patients are between 18 and 65 years old, understand Dutch, have no handicaps impeding participation and the principal DSM-IV-TR classification is undifferentiated somatoform disorder or chronic pain disorder. In contrast to other treatment effect studies, the co-morbidity of a personality disorder does not lead to exclusion. By this, we optimize the comparability between the study population and patients in daily practice enlarging the generalization possibilities.

Also in contrast to other effect studies, we chose quality of life (SF-36) instead of physical symptoms as the primary outcome measure. The SF-6D is used to estimate Quality Adjusted Life Years (QALYs). Costs are measured with the Trimbos/iMTA Questionnaire for Costs associated with Psychiatric Illness. Measurements are scheduled at baseline, after the training or waiting list, three and twelve months after the training. The differences between measurements are analyzed according to the intention-to-treat principle. The cost-effectiveness is expressed as costs per QALY, using multiple sensitivity analyses on the basis of a probabilistic model of the trial.

**Discussion:** If we show that our group training is (cost-)effective, more patients could be served, their quality of life could be improved while costs might be reduced. As the training is investigated in a heterogeneous patient group in the daily practice of a mental healthcare institution, its transfer to practice should be relatively easy.

**Trial registration:** Nederlands Trial Register, NTR1609

## Background

The estimated prevalence of unexplained physical symptoms in primary care ranges from 18 to 74% [1,2]. This huge difference in estimating prevalence is caused by multiple definitions of unexplained physical symptoms such as Unexplained Physical Symptoms (UPS), Medical Unexplained Physical Symptoms (MUPS), functional somatic syndromes and abridged somatization. We use the Diagnostic and Statistical Manual of Mental Disorders IV -Text Revision (DSM-IV-TR) [3] and define unexplained physical symptoms with the classification of 'undifferentiated somatoform disorder' and 'chronic pain disorder'. In the Netherlands, the estimated prevalence of unexplained physical symptoms in primary care is 18% [2]. Most of these unexplained physical symptoms can be classified as a somatoform disorder, of which 13.0% meets the criteria of undifferentiated somatoform disorder and 1.6% meets the criteria of chronic pain disorder [4]. In general, the DSM-IV-TR classifies symptoms without making assumptions about etiology. However, the DSM-IV-TR does presume psychological causes in the beginning, severity, increase or continuation of pain in chronic pain disorder. Therefore, we prefer to use the mere descriptive term 'Unexplained Physical Symptoms (UPS)', which corresponds with the DSM-IV-TR terms 'undifferentiated somatoform disorder' and 'chronic pain disorder' with the exception of the assumptions about etiology.

Patients with UPS have high levels of psychosocial distress and healthcare utilization [5], for which cognitive behavioral therapy is shown to be most effective [6-9]. However, it is widely believed, that patients with unexplained physical symptoms reject this kind of therapy. The consequences model is a positive exception. The key difference of this model compared to other cognitive behavioral models is its labeling of psychosocial distress in terms of consequences rather than as causes of UPS. Herewith, the consequences model fits both professionals' and patients' point of view. Refraining from labeling psychosocial distress as causes of UPS corresponds with the lack of consensus among professionals about the causes of UPS, which is reflected in the ongoing debate about the position of somatoform disorders on Axis I or III in the next edition of Diagnostic and Statistical Manual of Mental Disorders [10,11]. Moreover, labeling psychosocial distress as consequences, matches the patients' perspective of UPS. This is reflected in the fact, that 81% of the patients in academic medical care accepts an individual therapy based on the consequences model [12] and this individual therapy has positive outcomes in secondary care [13]. Unfortunately, in primary care, this high acceptance rate could not be reproduced and resulted in poor outcome [2,14].

We assume that the consequences model can maintain its positive outcome for patients in primary care, when we make some modifications. Firstly, we tailor this model more accurately to patients' perspective of their physical symptoms. Moreover, we put additional attention to relieve patients from being blamed for their symptoms. Furthermore, we make the group training easy accessible. Our aim is to investigate the (cost-)effectiveness of this easily accessible group training for patients in primary care conducted in the daily practice of our mental healthcare institution, Riagg Rijnmond, The Netherlands. This study protocol provides a detailed description of the cognitive behavioral group training and the design of the randomized controlled trial investigating the (cost-)effectiveness of this training.

### *Objectives*

The primary aim of this study is to investigate (cost-)effectiveness of our easily accessible protocollized group training for patients with UPS in primary care conducted in the daily practice of a mental healthcare institution. The secondary aim is to identify (personality-)variables enabling to predict this (cost-)effectiveness.

## Methods and design

### *Design*

The (cost-)effectiveness of the group training is evaluated in a randomized controlled trial (see Figure 1).

The study started February 2005. The inclusion of patients has ended in September 2008. The one-year follow-up period of the randomized patients will be finished in April 2010.

### *Study population*

Patients are included when:

1. they are between 18 and 65 years old;

2. they are able to speak, read and write Dutch;

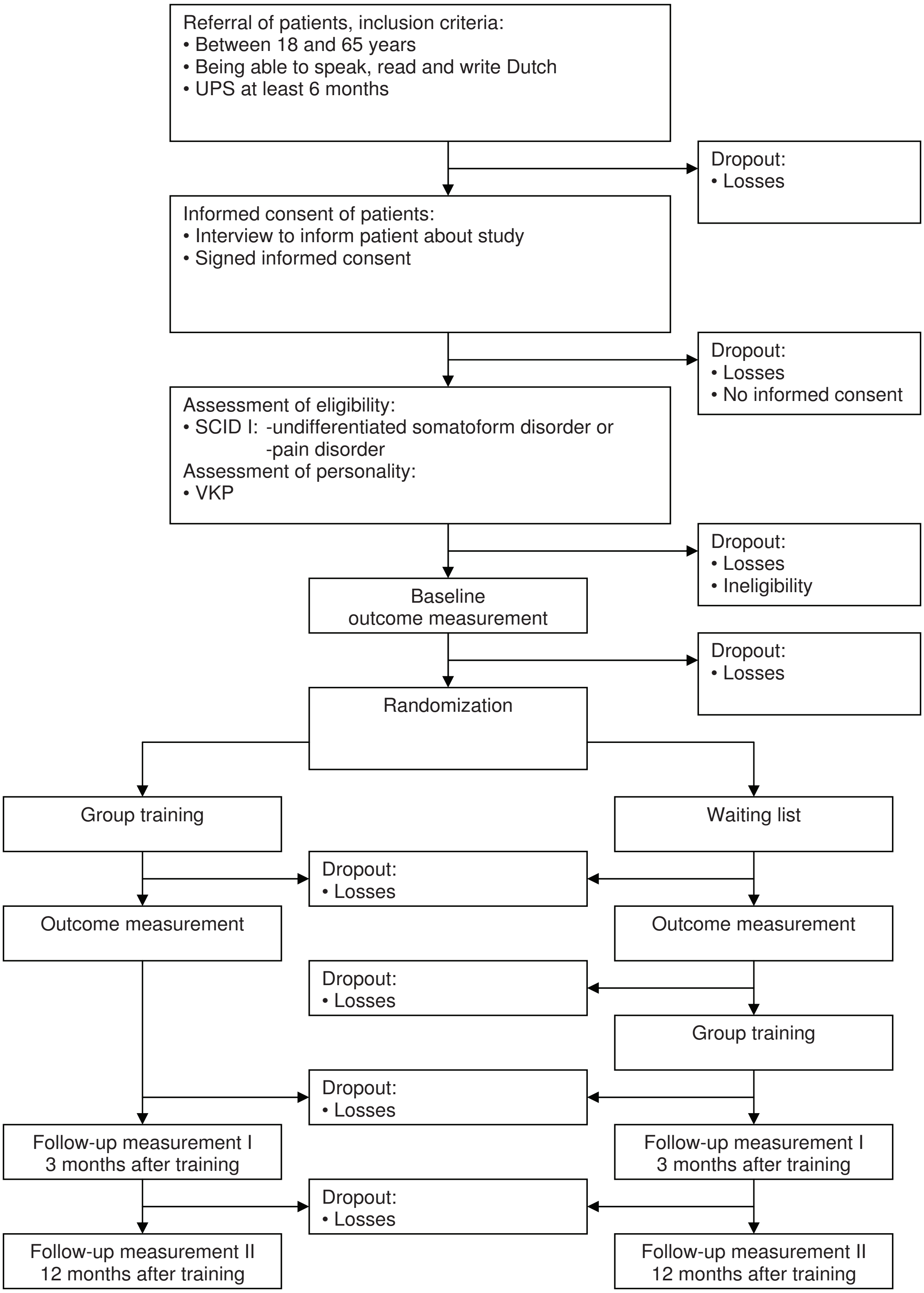


**Figure 1**
**Design of the randomized controlled trial.**

3. their UPS persists at least 6 months;

4. their UPS is classified as undifferentiated somatoform disorder or chronic pain disorder according to the criteria of the Structured Clinical Interview for DSM-IV Axis I Disorders/Patient edition (SCID-I/P) [15].

Patients are excluded when:

1. UPS is not the principal somatic disease;

2. undifferentiated somatoform disorder or chronic pain disorder is not the principal DSM-IV-TR classification;

3. handicaps like cognitive mental impairment and/or blindness impending the patient to participate in the training.

To optimize the comparability between the study population and the patients with UPS in daily practice and to make generalization to daily practice possible, we have decided that having a personality disorder is not an exclusion criterion; this is in contrast to other treatment effect studies. We do measure personality disorder with the self-report questionnaire for DSM-IV Axis II personality disorders (VKP) based on the International Personality Disorder Examination (IPDE) [16]. By measuring personality disorders using this instrument, we can not only describe the study population in terms of personality disorders, but also identify the influence of personality disorders on (cost-)effectiveness.

Patients are recruited from general practices and outpatient clinics of general hospitals in and nearby Rotterdam, the Netherlands. Physicians' attention is drawn to the group training by periodical postcards informing them when and how they can refer patients to the group training. Patients' attention is drawn to the group training by announcements in local newspapers and on websites of patients' associations, in which they are asked to make an appointment with their physician to discuss referral when interested. Physicians decide whether the physical symptoms are medically explained or unexplained hereby checking the first exclusion criterion, after which they refer patients when they find this appropriate. After referral, patients are invited for an interview, preferably in a medical setting, in which they are verbally and in writing informed about the study. In this interview, the first three inclusion criteria and the last exclusion criterion are verified. After receiving patients' signed informed consent, patients are invited for a second interview, in which the last inclusion criterion and the second exclusion criterion are investigated by the Structured Clinical Interview for DSM-IV Axis I Disorders/Patient edition (SCID-I/P) administered by independent psychologists. These psychologists make the final decision based on the results of the SCID-I/P whether patients' UPS can be classified as an undifferentiated somatoform disorder or as a chronic pain disorder and whether this disorder is the principal DSM-IV-TR classification. If undifferentiated somatoform disorder or chronic pain disorder is the principal DSM-IV-TR classification, then patients complete the self-report questionnaire for DSM-IV Axis II personality disorders (VKP).

Right before the start of each next training, the newly included patients fill in the questionnaires. Subsequently, an independent statistician randomizes them to either the group training or waiting list with a computer-based 1:1 ratio randomization procedure. The results of this randomization procedure are sent to the patient by letter. If randomization leads to starting with the group training, then an invitation for the group training is enclosed in the randomization letter. After the group training or after a waiting-period of the same length as the group training, all patients fill in the questionnaires for the second time.

After filling in the outcome measurements for the second time, the patients on the waiting list are invited to the group training. They follow the group training after their waiting period together with the newly included patients randomized to the group training in the most recent randomization. For patients on the waiting list, a longer waiting period is not feasible, because the study is conducted in the daily practice of a real life mental healthcare institution. By combining the patients assigned by the previous randomization to the waiting list with the patients assigned by the next randomization to the group training in the same training, the patients in both conditions receive exactly the same training. After completing the training, all patients fill in follow-up measurements after three months and after one year.

#### *Experimental condition: group training*

The group training [17] is based on the consequences model, which labels psychosocial stress as consequences rather than as causes of UPS to prevent the suggestion that 'its all in the head'. The original consequences model [18] assumes that UPS induces irrational beliefs regarding the symptoms resulting in consequences, which maintain or increase UPS (see black arrows in Figure 2). Its implementation in an individual therapy starts with beliefs labeling them as irrational, disputing them and replacing them with rational ones. Subsequently, other consequences are changed to break the vicious circle [19]. The ultimate goal is to reduce physical symptoms.

We assume that this original consequences model has not completely succeeded in preventing the 'its all in the head' suggestion. After all, patients might perceive the focus on irrational beliefs still as 'its all in the head'. Patients might

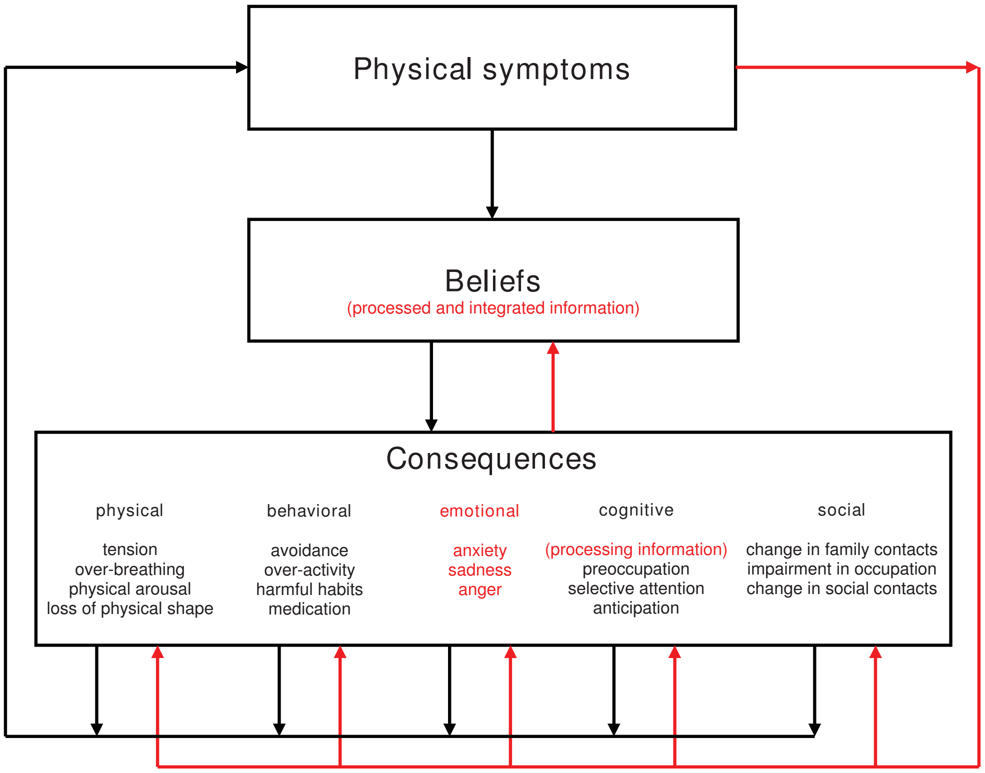


**Figure 2**
**Modified consequences model based on Speckens et al**. [18,19].

experience the disputing of these beliefs as blaming and belittling. Blaming and belittling result in a rejection of the therapy by patients and in poor outcome [20,21]. That is why we modify the consequences model for our group training (see red arrows in Figure 2). Our group training focuses on the visible consequences, labeling them as survival strategies in reaction to physical symptoms, justifying their existence by their benefit in short term, albeit indicating their harmfulness in the long run and therefore replacing them with long run beneficial strategies. Subsequently, the underlying beliefs of these survival strategies are explored, checked and, if necessary, changed in more helpful ones. Finally, the problem-solving model of Nezu *et al.* [22] is introduced to facilitate developing personal effective survival strategies for all kinds of problems, acknowledging that physical symptoms can increase the number of problems. The ultimate goal is not aimed at reducing physical symptoms as with the original consequences model, but is aimed at improving quality of life.

In summary, the group training uses the consequences model bottom-up instead of the commonly used top-down approach. By using the consequences model bottom-up (starting with consequences and unconditionally accepting and justifying their existence) instead of top-down (starting with addressing irrational beliefs and disputing them), we reach a closer match with the patients' physical point of view. Moreover, patients are relieved from being blamed, called exoneration, by justifying the existence of consequences by their benefit in short term, by which we facilitate compliance. Furthermore, we tailor the setting of the training to patients' physical point of view by organizing the training in a medical healthcare setting and not in our own mental healthcare institution. Herewith, also the implicit but unintended 'its all in the head' suggestion is avoided.

This bottom-up strategy results in a group training comprising thirteen ad verbatim protocollized weekly sessions of two hours each.

After session 1, the structure of each session is as follows:

• sharing experiences of the past week;

• discussing home-assignments;

• doing a group breathing and relaxation exercise;

• identifying short-term beneficial survival strategies and modifying them into long-term beneficial ones;

• ending with a summary of the session and new home-assignments.

Each session is built around a theme. In session 1, trainees get acquainted with each other by telling each other about their symptoms and by setting their personal goals for the training.

In session 2, the flight-fight cycle and habits in reaction to symptoms are identified as survival strategies of the body and modified with the learning of the breathing and relaxation exercise and the reshaping of habits into long-term beneficial ones.

In session 3, avoidance and overactivity in reaction to symptoms are identified as survival strategies of the body and modified by scheduling different kinds of activities in a feasible pace alternated with short breaks (5 to 10 minutes) that is compatible with the trainees' physical condition.

In session 4, emotions in reaction to symptoms are identified as useful survival strategies indicating the need for problem-solving. Moreover, the physical symptoms of emotions are notified and reduced or even relieved with the breathing and relaxation exercise.

In session 5, thoughts in reaction to symptoms are identified as survival strategies of the mind and, if necessary, modified with the Ellis' ABCDE scheme into more helpful ones.

In session 6, a good physical shape is identified as an effective survival strategy of the body, which can be achieved by doing a low cardiac physical activity (like walking or biking) twice a day and increasing this up to a maximum of 60 minutes twice a day, after which physical shape can be maintained by a regular sport twice a week.

In session 7, the form at Figure 2 is filled in by the trainees and discussed afterwards with an important and trusted person in his or her own social environment.

In sessions 8 to 12, the five steps of the problem-solving method (problem-attitude, problem-definition, alternative solutions, solution plan, solution implementation and evaluation) are identified and practiced.

In session 13, a personal First Aid Kit is composed out of the learned long-term beneficial survival strategies aimed to prevent relapse.

#### *Control condition: waiting list*
Patients assigned to the waiting list condition, wait during the group training of 13 weeks, after which they start with their training.

#### *Outcome measurements*
(Cost-)effectiveness is measured with three self-report questionnaires, which are sent to patients' home to be completed before randomization, at the end of the training or waiting list period, after 3 and 12 months after the training (see Figure 1).

1) Short Form Health Survey (SF-36) [23]

The SF-36 measures functional health and well-being during the past four weeks with the following eight multi-item scales: Physical functioning, Role functioning physical, Bodily pain, General health, Vitality, Social functioning, Role functioning emotional and Mental health. The scores of the SF-36 can also be summarized in the Mental and the Physical summary scores [24]. Furthermore, a utility score can be derived from 11 items of the SF-36. These 11 items define six dimensions of health, the SF-6D; Physical functioning, Role limitations (Role functioning physical in combination with Role functioning emotional), Bodily pain, Social functioning, Vitality and Mental health. The outcome of the SF-6D can be converted into Quality Adjusted Life Years (QALYs), the preferred outcome in health economics, using formerly called 'valuations studies' [25].

2) Symptom Checklist Revised (SCL-90-R) [26]

The SCL-90-R measures a broad range of symptoms and their intensity during the past week with the following eight multi-item scales: Phobic anxiety, Anxiety, Depression, Somatization, Obsessive-compulsive, Interpersonal sensitivity, Hostility and Sleep difficulties. The scores of the SCL-90-R can be summarized in the Global severity index, reflecting the overall psychological distress.

3) Trimbos/iMTA Questionnaire for Costs associated with Psychiatric Illness (TiC-P) [27]

The TiC-P measures direct medical costs due to healthcare utilization during the past four weeks, excluding the group training itself. The costs of the training itself are calculated using the records of the institution. The TiC-P also registers the indirect non-medical costs due to productivity loss during the past two weeks. This second part of the questionnaire about indirect costs is based on the short form of the Health and Labour Questionnaire (HLQ).

#### *Outcome measurements: clinical evaluation*
The aim of the clinical evaluation is to investigate the effectiveness of the group training by comparing the improvement of quality of life gained in the training group to the one in the waiting list group. Primary outcome measure is the Mental and Physical summary score of the SF-36. Secondary outcomes are the eight individual scales of the SF-36 and the scales of the SCL-90-R.

#### *Outcome measurements: economic evaluation*
The aim of the economic evaluation is to investigate cost-effectiveness of the group training in terms of cost per Quality Adjusted Life Years (QALYs). QALYs are estimated by converting SF-6D into utilities by means of the preference-based UK tariff [25]. Indirect costs for employed patients are measured with the TiC-P by the reported duration of sick leave and the production loss without sick leave. The indirect costs of production loss due to sick leave are computed by multiplying the number of sick leave's day with the average net income per worker related to age and gender. By a long-term sick leave, the friction-cost method is applied to assess the productivity loss, using a friction period of 5 months.

#### *Sample size calculation*
To determine the required sample size for measuring differences in quality of life between the two conditions (group training and waiting list), the sample size is calculated by power analysis. The effect size of cognitive behavioral therapy for quality of life is not well known, because in other effect studies the outcome is usually measured in terms of physical symptoms. Therefore, we have to use effect size of cognitive behavioral therapy for physical symptoms as an available estimator for the (shortage of) quality of life. The effect size of cognitive behavioral therapy for physical symptoms ranges from .00 to .95 [8], suggesting a medium effect for cognitive behavioral therapy for physical symptoms. Assuming this effect also applies for quality of life, the magnitude of the effect size following Cohen's (1988) [28] is 0.50. With a power of .80 and an alpha of .05 (two-tailed), a sample size of 100 patients (50 in each condition) is required.

#### *Statistical analyses*
The comparability of the patients' baseline-variables between the two conditions (group training and waiting list) is analyzed with the two-tailed t-tests for independent samples for the continuous variables, with the two-tailed Mann-Whitney U-tests for the ordinal variables and with the chi-square tests for the categorical variables. If the patients in the two conditions are not comparable on one or more baseline-variables, those variables will be utilized as covariables in the subsequent analyses.

#### *Statistical analyses: clinical evaluation*
The clinical evaluation is conducted according to the intention-to-treat principle. The effectiveness of the group training for primary and secondary outcome measures is analyzed with mixed modeling (i.c. random regression modeling). Baseline measurements, corresponding to the subsequent outcome measurements, are entered as covariables. This method of mixed modeling for repeated measurements enables the use of flexible error covariance structures. In addition, the predictive performance of baseline-variables, especially personality variables, on effectiveness can be estimated.

#### *Statistical analyses: economic evaluation*
The economic evaluation is conducted from a societal perspective, the preferred perspective in health economic evaluations [29]. This means that all costs are included: the direct medical costs, the indirect medical costs and the indirect costs associated with productivity loss of patients. Adopting a societal perspective also means that all relevant effects and all costs beyond the time frame of the trial should be measured. In this case, the differences between group training and waiting list can only be measured empirically till 13 weeks, but relevant effects and costs might occur beyond that artificial time horizon. For this reason, we will estimate effects and costs till 2 years, using a Markov model [30]. By making the Markov model probabilistic, we will be able to implement multiple sensitivity tests simultaneously and test for specific model assumptions. A critical assumption will be the extrapolation of the effect beyond 13 weeks. This assumption will be explored by calculating the minimal duration that the group training should be effective, in order to achieve a satisfactory level of cost-effectiveness.

## Ethical considerations
The Medical Ethical Committee of Erasmus Medical Center, Rotterdam, The Netherlands, has approved this study, registered under MEC-2004-191.

## Discussion
The primary aim of our study is to investigate (cost-)effectiveness of an easily accessible protocollized group training for patients with UPS in primary care in the daily practice of our mental healthcare institution, Riagg Rijnmond, The Netherlands.

Investigating the (cost-)effectiveness of our training in the daily practice of a mental healthcare institution has its benefits and its limitations. The huge advantage of investigating the (cost-)effectiveness in the daily practice is that if (cost-)effectiveness is shown, the group training can be started without the delay of practical implementation issues. The limitations for this study are less possibilities for exclusion of patients and for the control condition.

Consequently, the (cost-)effectiveness is explored in a heterogeneous group of patients. Measuring heterogeneity of our study population with the SCID-I/P and VKP but not excluding co-morbidity and drawing attention to the group training by announcements to both physicians and patients acumilate this heterogeneity. Patients being referred on their own initiative are probably more motivated than patients being referred on their physician's initiative. On the one hand, this enhances the probability of a representative study population, whose heterogeneity will be equally divided between the two conditions by randomization. Furthermore, this heterogeneous study population is real practice, making the study results realistic estimates of that practice. On the other hand, the group training might be effective by only treating the co-morbidity, like anxiety. Analyzing the predictability of this co-morbidity and other baseline variables on (cost-)effectiveness can solve this limitation.

Because the daily practice only allows a waiting list for a short period, the follow-up measurements do not have a control condition. Repeated measurements in the same patients and using a probabilistic model can solve this problem partly.

If we show that our group training is feasible in daily practice and (cost-)effective, more patients with UPS could be served, their quality of life could be improved while costs might be decreased.

## Abbreviations used

UPS: Unexplained Physical Symptom(s); DSM-IV-TR: Diagnostic and Statistical Manual of Mental Disorders IV -Text Revision; SCID-I/P: Structured Clinical Interview for DSM-IV Axis I Disorders/Patient edition; VKP: Self-report questionnaire for DSM-IV Axis II personality disorders; IPDE: International Personality Disorder Examination; SF-36: Short Form Health Survey; SF-6D: Short Form 6 Dimensions; SCL-90-R: Symptom Checklist Revised; TiC-P: Trimbos/iMTA Questionnaire for Costs associated with Psychiatric Illness; HLQ: Health and Labour Questionnaire.

## Competing interests

The authors declare that they have no competing interests.

## Authors' contributions

LNLZ developed the original idea of the study, wrote the ad verbatim manual for the group training, implemented the study in the daily practice of the real life mental healthcare institution, Riagg Rijnmond, The Netherlands. HJD substantially contributed to the randomization and the statistical methods. JJvB substantially contributed to the economic evaluation methods. All authors read and corrected draft versions. They approved the final version of this manuscript.

## Acknowledgements

The first author is indebted to Riagg Rijnmond, The Netherlands. This study could not have been designed without the colleagues, financial support and facilities of Riagg Rijnmond. Her considerable appreciation and gratefulness go to the late H. Methorst, psychiatrist and psychoanalyst at Riagg Rijnmond, whose trust in and support to the author she will always remember. The authors' considerable appreciation and gratefulness also go to the late professor R.W. Trijsburg PhD, who supervised the development and start of the study.

## References

1. Kroenke K: **Patients presenting with somatic complaints: Epidemiology, psychiatric co-morbidity and management.** *International Journal of Methods in Psychiatric Research* 2003, **12(1):**34-43.
2. Arnold IA, Speckens AEM, Van Hemert AM: **Medically unexplained physical symptoms: The feasibility of group cognitive-behavioural therapy in primary care.** *J Psychosom Res* 2004, **57(6):**517-520.
3. APA: *Beknopte handleiding bij de diagnostische criteria van de DSM-IV-TR* second edition. Lisse: Harcourt Assessment BV; 2005.
4. de Waal MWM, Arnold IA, Eekhof JAH, van Hemert AM: **Somatoform disorders in general practice: Prevalence, functional impairment and comorbidity with anxiety and depressive disorders.** *Br J Psychiatry* 2004, **184(6):**470-476.
5. Barsky AJ, Orav EJ, Bates DW: **Distinctive patterns of medical care utilization in patients who somatize.** *Med Care* 2006, **44(9):**803-811.
6. Kroenke K: **Efficacy of treatment for somatoform disorders: A review of randomized controlled trials.** *Psychosom Med* 2007, **69(9):**881-888.
7. Sumathipala A: **What is the evidence for the efficacy of treatments for somatoform disorders? A critical review of previous intervention studies.** *Psychosom Med* 2007, **69(9):**889-900.
8. Nezu AM, Nezu CM, Lombardo ER: **Cognitive-behavior therapy for medically unexplained symptoms: a critical review of the treatment literature.** *Behavior Therapy* 2001, **32(3):**537-583.
9. Looper KJ, Kirmayer LJ: **Behavioral Medicine Approaches to Somatoform Disorders.** *J Consult Clin Psychol* 2002, **70(3):**810-827.
10. Kroenke K, Sharpe M, Sykes R: **Revising the classification of somatoform disorders: Key questions and preliminary recommendations.** *Psychosomatics: Journal of Consultation Liaison Psychiatry* 2007, **48(4):**277-285.
11. Fink P, Rosendal M: **Recent developments in the understanding and management of functional somatic symptoms in primary care.** *Current Opinion in Psychiatry* 2008, **21(2):**182-188.
12. Speckens AEM, Van Hemert AM, Bolk JH, Hawton KE, Rooijmans HGM: **The acceptability of psychological treatment in patients with medically unexplained physical symptoms.** *J Psychosom Res* 1995, **7:**855-863.
13. Speckens AEM, Van Hemert AM, Spinhoven P, Hawton KE, Bolk JH, Rooijmans HGM: **Cognitive behavioural therapy for medically unexplained physical symptoms: a randomised controlled trial.** *Br Med J* 1995, **311:**1328-1332.
14. Arnold IA, De Waal MWM, Eekhof JAH, Spinhoven P, Assendelft WJJ, Van Hemert AM: **Onverklaarde lichamelijke klachten en de samenhang met depressie en angst.** *Huisarts & Wetenschap* 2007, **50(13):**686-689.
15. First MB, Spitzer RL, Gibbon M, Williams JBW: *Structured Clinical Interview for DSM-IV Axis I Disorders* Amsterdam: Harcourt Test Publishers; 1999.
16. Duijsens IJ, Haringsma R, Eurelings-Bontekoe EHM: *V.K.P. Handleiding* Leiderdorp: Datec; 2002.
17. Zonneveld LNL: **Draaiboek 'Omgaan met de gevolgen van medisch onverklaarde lichamelijke klachten'.** Vlaardingen/Rotterdam: Riagg Rijnmond/Erasmus Medical Center, Department of Medical Psychology and Psychotherapy; 2005.
18. Speckens AEM, Spinhoven P, Hawton KE, Bolk JH, Van Hemert AM: **Cognitive behavioural therapy for medically unexplained**

**physical symptoms: A pilot study.** *Behavioural and Cognitive Psychotherapy* 1996, **24(1):**1-16.
19. Speckens AEM, Spinhoven P, Van Rood YR: **Protocollaire behandeling van patiënten met onverklaarde lichamelijke klachten: Cognitieve gedragstherapie.** In *Protocollaire behandelingen in de ambulante geestelijke gezondheidszorg 2* Edited by: Keijsers GPJ, Van Minnen A, Hoogduin CAL. Houten/Diegem: Bohn Stafleu Van Loghum; 1999:199-236.
20. Lambert MJ: **Early response in psychotherapy: Further evidence for the importance of common factors rather than "placebo effects".** *J Clin Psychol* 2005, **61(7):**855-869.
21. Watson JC, Geller SM: **The relation among the relationship conditions, working alliance, and outcome in both process-experiential and cognitive-behavioral psychotherapy.** *Psychotherapy Research* 2005, **15(1–2):**25-33.
22. Nezu AM, Nezu CM, Friedman SH, Faddis S, Houts PS: *Helping cancer patients cope: a problem-solving approach* Washington, DC, US: American Psychological Association; 1998.
23. Ware J, Snow KK, Kosinski M, Gandek B: **How to score the SF-36 Health Survey.** In *SF-36 Health Survey: Manual and Interpretation Guide* Boston, MA: The Health Institute, New England Medical Center; 1993.
24. Ware JE, Kosinski M, Keller SD: *SF-36 Physical and Mental Health Summary Scales: A User's Manual* Boston, MA: The Health Institute; 1994.
25. Brazier J, Roberts J, Deverill M: **The estimation of a preference-based measure of health from the SF-36.** *J Health Econ* 2002, **21(2):**271-292.
26. Arrindell WA, Ettema JHM: *SCL-90: handleiding bij een multidimensionele psychopathologie-indicator* Lisse: Swets Test Publishers; 1986.
27. Hakkaart-van Roijen L: *Handleiding Trimbos/iMTA questionnaire for Costs associated with Psychiatric Illness (TiC-P). juli 2002 edition* Rotterdam: Institute for Medical Technology Assessment & Erasmus Universitair Medisch Centrum Rotterdam; 2002.
28. Cohen J: *Statistical power analysis for the behavioral sciences* Hillsdale, New York: Lawrence Erlbaum; 1988.
29. Drummond MF, Sculpher MJ, Torrance GW, O'Brien BJ, Stoddart GL: *Methods for the Economic Evaluation of Health Care Programmes* Third edition. Oxford, England: Oxford University Press; 2005.
30. Briggs A, Sculpher M, Claxton K: *Decision Modelling for Health Economic Evaluation* Oxford, England: Oxford University Press; 2006.

## Pre-publication history

The pre-publication history for this paper can be accessed here:

http://www.biomedcentral.com/1471-2458/9/251/prepub

Publish with BioMed Central and every scientist can read your work free of charge

"BioMed Central will be the most significant development for disseminating the results of biomedical research in our lifetime."

Sir Paul Nurse, Cancer Research UK

Your research papers will be:

- available free of charge to the entire biomedical community
- peer reviewed and published immediately upon acceptance
- cited in PubMed and archived on PubMed Central
- yours — you keep the copyright

Submit your manuscript here:
http://www.biomedcentral.com/info/publishing_adv.asp

BioMedcentral
